# Dental caries and externalizing behaviour problems in a high‐risk child population

**DOI:** 10.1111/eos.12542

**Published:** 2018-07-27

**Authors:** Maddelon de Jong‐Lenters, Denise Duijster, Annemarie Schuller, Cor van Loveren, Erik Verrips

**Affiliations:** ^1^ TNO, Netherlands Organisation for Applied Scientific Research Leiden The Netherlands; ^2^ Department of Cariology Endodontology Pedodontology Academic Centre for Dentistry Amsterdam University of Amsterdam and VU University Amsterdam The Netherlands; ^3^ Department of Social Dentistry Academic Centre for Dentistry Amsterdam University of Amsterdam and VU University Amsterdam The Netherlands; ^4^ Centre for Dentistry and Oral Hygiene (CTM) University Medical Center Groningen Groningen The Netherlands; ^5^ Department of Preventive Dentistry Academic Centre for Dentistry Amsterdam University of Amsterdam and VU University Amsterdam the Netherlands

**Keywords:** dental caries, oral health, parenting, problem behaviour

## Abstract

The aim of this study was to assess the association between externalizing behaviour problems and dental caries in children. A further objective was to explore direct and indirect pathways between sociodemographic factors, family functioning and parenting factors, oral health behaviours, externalizing behaviour problems, and dental caries using structural equation modelling. Cross‐sectional data were collected on 251, 5‐ to 8‐yr‐old children from a paediatric dental practice in the Netherlands. Children's decayed, missing, and filled primary teeth (dmft) scores were obtained from their dental records. Validated self‐report questionnaires were used to collect sociodemographic, behavioural, and family‐related data. Externalizing problem behaviour was significantly associated with a higher dmft score [incidence risk ratio (IRR) = 1.19; 95% CI: 1.06–1.34], but this association did not remain significant after adjustment for sociodemographic factors (IRR = 1.11; 95% CI: 0.99–1.26). A valid path model was presented after applying some modifications. Findings from the model suggest that it is plausible that child behaviour problems are directly associated with dental caries via toothbrushing behaviour. The model also provided support that maternal education level, the restrictiveness and warmth of parenting, and the communication of the family, play an indirect role in the association between children's externalizing behavioural problems and dental caries experience.

Externalizing behaviour problems have been increasingly diagnosed in children in developed countries 1, 2. Examples of externalizing behaviour problems are attention‐deficit hyperactivity disorder (ADHD), temperament, impulsiveness, and general conduct problems 3. There is evidence to suggest that externalizing child behaviour problems and dental caries are related. williamson *et al*. found that externalizing behaviour problems were significantly more prevalent in caries‐active children than in caries‐free children 4. Greater dental caries experience has been reported in children with ADHD 5, 6, yet other studies have claimed that the levels of caries are actually lower in this group of children 7, 8.

There are several plausible explanations for the relationship being positive. A possible *direct* explanation is that good oral hygiene and limiting the intake of sugary snacks is more challenging in children with externalizing problem behaviour 7 because such children potentially show lower levels of compliance. *Indirectly*, the family environment could be an underlying influential factor on both children's problem behaviour and the adoption of children's health behaviours 9. Family interactions, such as expression of affection, parents’ discipline practices, and family routines, provide the context in which parents’ behavioural directions are delivered to and interpreted by the child. There are several aspects of the family environment (i.e. parenting and family functioning) that have been associated with negative child outcomes, such as an unhealthy diet, dental caries, and behaviour problems in children 10, 11, 12. Parenting has been described as a versatile and complex behavioural pattern consisting of two dichotomies: warmth vs. hostility; and restrictiveness vs. autonomy 13. Family functioning is a broader concept that describes how the interactions between all family members affect the way in which both children and parents manage daily life 14. Greater dental caries experience was found in children of families with ineffective parenting, characterized by high levels of demand, along with low levels of positive involvement and encouragement 15, 16, and in children of poor functioning families in terms of inadequate communication, low levels of responsiveness, and poor organization 17, 18. Similar aspects of parenting and family functioning have also been associated with an increased risk of externalizing behaviour problems, in addition to aspects such as high levels of conflict and harsh discipline practices 12, 14. It has not yet been investigated whether certain aspects of the family environment are common determinants for the development of both dental caries and externalizing behaviour problems in children.

In summary, the literature on the relationship between child externalizing behaviour problems and dental caries is contradictory, and therefore inconclusive. In addition, the underlying mechanisms of this potential relationship remain unclear. Therefore, the primary aim of this study was to assess the association between externalizing behaviour problems and dental caries in children. A further objective was to explore direct and indirect pathways between sociodemographic factors, family functioning and parenting factors, oral health behaviours, externalizing behaviour problems, and dental caries in children using structural equation modelling.

## Material and methods

Approval for this study was obtained from the Ethics Committee for patient‐related research of the VU University (VU METC, nr 2012/393). All parents signed written consent forms before their children were included in the study.

### Study sample

The data for this study were collected in a referral centre for paediatric dental care in Noordwijk (the Netherlands), to which children are referred for various diagnoses, including early childhood caries in very young children, congenital dental disorders, psychological problems, behaviour management problems, dental fear, and developmental problems. A small percentage of the children have special needs associated with physical or learning difficulties. The ages of children referred to the centre vary widely. For the purposes of this study, all children between 5 and 8 yr of age were selected from the referral centre's patient population. Children with special needs were excluded. Because the aim of the study was exploratory, no a priori hypotheses were put forward on the size of putative associations and, as a consequence, no power calculations were performed.

An invitation letter with information about the study, an informed consent form, and a questionnaire were sent to the children's parents. To increase the response rate, a prepaid return envelope was attached and participants received a monetary incentive (€10). Non‐respondents were first sent a reminder by post with another copy of the questionnaire after 4 wk, followed by a reminder by telephone after an additional 4–6 wk.

### Data collection

Data on dental caries were obtained from electronic patient records at the paediatric dental centre. The diagnosis of dental caries was based on clinical examinations supported by dental radiographs, mostly bitewings, on the condition that the patient cooperated. The dental examinations and interpretation of dental radiographs were performed by two paediatric dentists working at the centre. They used protocolled procedures for recording diagnosed dental caries lesions and reasons for restorations or extractions in the electronic patient record, every time that patients attended the centre. Children's dmft scores [i.e. the sum of decayed (d), missing (m), and filled (f) deciduous teeth] were obtained from these dental health records, using data of the children's most recent visit to the paediatric dental centre. Missing teeth were not scored if they were absent as a result of dental trauma, hypomineralization, agenesis, or normal exfoliation; they were only scored if records indicated that they were extracted because of caries.

Externalizing behaviour problems in children were scored by combining the ‘hyperactivity‐inattention’ and ‘conduct problems’ domains of the Strengths and Difficulties Questionnaire (SDQ) 19. This is a concise questionnaire that has proven of value, over time, to measure psychosocial adjustment in children and adolescents. The parental version for children aged 4–17 yr was used in this study. Both subscales consist of five items with answers on a three‐point Likert scale (0 being ‘not true’, 1 being ‘somewhat true’, and 2 being ‘certainly true’). An example of an item used to measure ‘conduct problems’ is ‘My child often lies or cheats’; an example of an item measuring ‘hyperactivity‐inattention’ scale is ‘My child is easily distracted, concentration wanders’. The SDQ scores for both subscales were categorized into average, elevated, and high using normative cut‐off points from the original Dutch SDQ 20. Given the low number of children allocated to the high and elevated categories, these were combined into one category: elevated/high. Subsequently, children were grouped as having externalizing behaviour problems if they had elevated or high scores for hyperactivity‐inattention and/or conduct problems.

A parental‐administered questionnaire was used to collect information about sociodemographic variables, such as the child's date of birth, gender, the mother's country of birth, and the mother's highest completed level of education. The mother's country of birth was categorized into the Netherlands or any other country. There were three categories of educational level: (i) lower education (no education, elementary school, and lower general education); (ii) intermediate education (higher general education and lower vocational education); and (iii) higher education (higher vocational education or university). Oral health behaviours were measured using two items: the age at which toothbrushing was started (<1 yr, 1–2 yr, more than 2 yr); and the frequency of toothbrushing per day (once or less per day, twice or more per day).

The Gezinsvragenlijst (GVL; translation Family Questionnaire) is a validated instrument used to measure the quality of family and parenting circumstances in children aged 4–18 yr 14. Among other domains, the GVL measures responsiveness, communication, and organization, which have been shown to be related to children's oral health behaviours, dental caries, and child behaviour problems. The three subscales each consist of nine items with answers given on a five‐point Likert scale ranging from ‘strongly agree’ to ‘strongly disagree’. An example of an item measuring responsiveness is ‘We give our child a lot of compliments’; an example of an item covering communication scale is ‘We find it hard to understand our child’; one item used to measure organization is ‘We strive for order and regularity in our household’. All nine answers in each subscale were summed, resulting in subscale scores ranging from 9 to 45. Higher scores indicate poorer functioning. Each subscale is subsequently classified into normal, subclinical, and clinical family functioning using normative cut‐off scores provided by the authors of the instrument 14. When a value for one item was missing, the mean score for the remaining eight items of that subscale was calculated and added to the total score of that subscale. This was the case in 4.4% of the responses for the subscale responsiveness and in 4.8% of the responses for the subscale organization.

The Child Rearing Practices Report (CRPR) was used to assess the norms, values, attitudes, behaviours, and intent of either maternal or paternal parenting. This instrument has proven reliability and construct validity over time 21. Of the 40 items in total, 22 measure the restrictiveness of parents (for example: ‘I do not accept my child getting angry at me’). The other 18 items assess nurturance (example being ‘I think you should comfort a child when it is upset’). As no normative scores have been published for the CRPR, the total scores obtained with the two subscales of the CRPR were classified as low or high on the basis of the median of the two scale scores in the current study. If there were one or two missing items in one subscale, the mean subscale score was imputed. This was the case in 1.6% of the responses for the parenting subscales. Table 1 presents an overview of the family domains measured in this study, including a definition of each domain, the number of items per subscale, and internal consistency.

**Table 1:** Definition, number of items, and internal consistency for social–behavioural constructs

Constructs	Definition	No. of items	Cronbach's *α*
Family functioning (GVL)			
Responsiveness	To what extent parents respond to the needs of their child in different personal and cognitive fields.	9	0.75
Communication	Parent–child interaction: the degree they listen to each other and respond to each other's needs in harmonious and less harmonious situations.	9	0.76
Organization	The degree of structure, routines, and assignment of roles in the family, as well as the family's ability to resolve problems.	9	0.75
Parenting (CRPR)			
Restrictiveness	Parents’ tendency to confine behaviour within certain specified limits.	22	0.84
Nurturance	Interaction between parent and child in which parent responds to the child's needs.	28	0.81

CRPR, Child Rearing Practices Report 21; GVL, Gezinsvragenlijst (Family Questionnaire) 14.

### Statistical analysis

STATA version 15 (Stata, College Station, TX, USA) was used for statistical analysis. In all hypothesis‐testing, a test result for which a value of *P* < 0.05 was obtained was considered to indicate a statistically significant deviation from the null hypothesis. As the dmft was a non‐normally distributed count variable, Poisson regression was used to assess the association between externalizing behaviour problems and dmft. The association was subsequently adjusted for age, gender, and the mother's education level and country of birth, to correct for potential confounders. Univariate analyses were performed to assess whether sociodemographic variables, oral health behaviours, family functioning, and parenting variables were associated with dmft (Poisson regression) and with externalizing behaviour problems (logistic regression).

**Figure 1:**
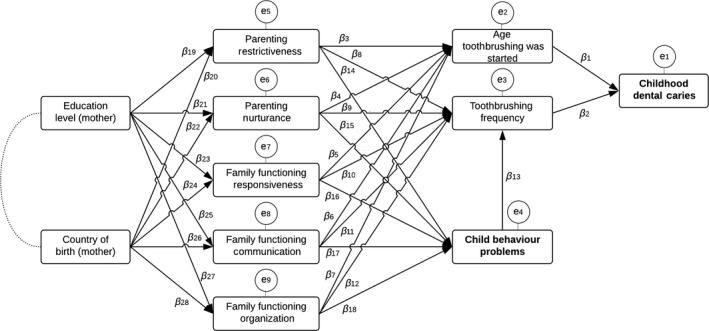
Schematic illustration of the hypothesized path model. Arrows imply that a variable has an influence on another variable; round connecting lines imply that variables are associated. The a priori hypotheses of the path model are as follows.

Structural equation modelling was used to test the fit of an a priori hypothesized path model of direct and indirect pathways between sociodemographic variables, family functioning and parenting variables, oral health behaviours, child behaviour problems, and dmft. The hypothesized path model is shown in Fig. 1, including the a priori hypotheses. All variables in the path model were included as categorical variables, except for the count variable dmft. Unstandardized and standardized path coefficients were reported. The following goodness‐of‐fit measures were assessed to determine the adequacy of model fit to the data: the *χ* ^2^/d.f. ratio and its *P*‐value, the root mean square error of approximation (RMSEA), the standardized root mean square residual (SRMR), and the comparative fit index (CFI). Good fit was indicated by a nonsignificant *χ* ^2^ value, RMSEA and SRMR values below 0.07, and a CFI value greater than 0.95. In the event of poor fit, modifications to the model were explored to improve model fit, based on inspection of the standardized residual matrix and the statistical significance of regression coefficients. There were missing values for some of the variables, varying from *n *=* *1 to *n *=* *21. Complete case analysis was used to handle missing data.

- dmft = (*β*_1_ • age toothbrushing was started) + (*β*_2_ • toothbrushing frequency) + *e*_1_.
- Age toothbrushing was started = (*β*_3_ • parenting restrictiveness) + (*β*_4_ • parenting nurturance) + (*β*_5_ • family functioning responsiveness) + (*β*_6_ • family functioning communication) + (*β*_7_ • family functioning organization) + *e*_2_.
- Toothbrushing frequency = (*β*_8_ • parenting restrictiveness) + (*β*_9_ • parenting nurturance) + (*β*_10_ • family functioning responsiveness) + (*β*_11_ • family functioning communication) + (*β*_12_ • family functioning organization) + (*β*_13_ • externalizing behaviour problems) + *e*_3_.
- Externalizing behaviour problems = (*β*_14_ • parenting restrictiveness) + (*β*_15_ • parenting nurturance) + (*β*_16_ • family functioning responsiveness) + (*β*_17_ • family functioning communication) + (*β*_18_ • family functioning organization) + *e*_4_.
- Parenting restrictiveness = (*β*_19_ • education level (mother)) + (*β*_20_ • country of birth (mother)) + *e*_5_.
- Parenting nurturance = (*β*_21_ • education level (mother)) + (*β*_22_ • country of birth (mother)) + *e*_6_.
- Family functioning responsiveness = (*β*_23_ • education level (mother)) + (*β*_24_ • country of birth (mother)) + *e*_7_.
- Family functioning communication = (*β*_25_ • education level (mother)) + (*β*_26_ • country of birth (mother)) + *e*_8_.
- Family functioning organization = (*β*_27_ • education level (mother)) + (*β*_28_ • country of birth (mother)) + *e*_9_.

## Results

Of the 450 families approached, 55.7% returned the questionnaire. The majority of questionnaires were completed by the mother (*n* = 227; 90.4%) and the remaining 24 (9.6%) questionnaires were completed by the father. The study sample consisted of 251 children; just over half (50.6%) were girls. The mean age of the children was 6.6 yr ± 0.8 (mean ± SD) on the date of completing the questionnaire. The mean dmft of children was 4.6 ± 3.2 (range: 0–12) and only 15.9% of the children in the sample had a dmft of 0. Of the 251 children, 107 (43.7%) had elevated or high scores for externalizing behaviour problems. Table 2 describes the distribution of sociodemographic variables, oral health behaviours, and family functioning and parenting variables in the study sample.

**Table 2:** Description of the study sample

**Characteristics**	**Value**
Age (yr)	6.6 ± 0.8 (4.6–8.5)
Gender	
Boy	124 (49.4)
Girl	127 (50.6)
Educational level (mother)	
Lower education	62 (24.7)
Intermediate education	119 (47.4)
Higher education	70 (27.9)
Country of birth (mother)	
The Netherlands	208 (82.9)
Other	43 (17.1)
Age toothbrushing was started	
<1 yr	138 (56.1)
1–2 yr	89 (36.2)
>2 yr	19 (7.7)
Toothbrushing frequency	
1 time or less a day	63 (25.2)
2 times a day or more	187 (74.8)
Family functioning – responsiveness	
Normal	230 (91.3)
Subclinical	19 (7.5)
Clinical	3 (1.2)
Family functioning – communication	
Normal	204 (82.6)
Subclinical	32 (13.0)
Clinical	11 (4.5)
Family functioning – organization	
Normal	206 (82.4)
Subclinical	32 (12.8)
Clinical	12 (4.8)
Parenting – restrictiveness	
Low	116 (50.4)
High	114 (49.6)
Parenting – nurturance	
Low	128 (51.6)
High	120 (48.4)

Data are given as mean ± SD (range) or *n* (%).

The mean dmft in children with elevated/high scores for externalizing behaviour problems was 4.9 ± 3.1, compared with 4.2 ± 3.2 in children with average scores. Poisson regression showed that children with elevated/high scores for behaviour problems had 19% greater caries experience than children with average scores for behaviour problems [incidence risk ratio (IRR) = 1.19, 95% CI: 1.06–1.34, *P *=* *0.004]. This association did not remain statistically significant after adjustment for child's age, gender, and the mother's education level and country of birth (IRR = 1.11, 95% CI: 0.99–1.34, *P *=* *0.080).

Table 3 shows the univariate associations of sociodemographic, behavioural, and family‐related factors with dmft and with child behaviour problems. Children whose mother had a higher level of education had significantly lower levels of dmft, while immigrant children had a significantly higher dmft. Children who started toothbrushing after the age of 2 yr had 33% higher levels of dmft than children who started toothbrushing before they were 1 yr old. The frequency of toothbrushing was not significantly associated with mean dmft. Children from poor functioning families (clinical scores) in terms of responsiveness, communication, and organization, and children of parents with restrictive parenting behaviours, had a significantly higher dmft. No statistical association between parenting in terms of nurturance and dmft was found. With respect to externalizing behaviour problems, no significant associations with the mother's education level, the mother's country of birth, and toothbrushing behaviours were found. Children from poor functioning families in terms of communication and children of parents who reported low levels of nurturance were significantly more likely to have ‘high/elevated’ scores for externalizing behaviour problems. Externalizing behaviour problems were not associated with family functioning in terms of responsiveness and organization, or with restrictive parenting.

**Table 3:** Univariate associations between sociodemographic, behavioural, and family‐related factors and decayed, missing, and filled primary teeth (dmft; Poisson regression) and externalizing behaviour problems (logistic regression)

	dmft		Externalizing behaviour problems	
	IRR (95% CI)	*P*‐value^a^	OR (95% CI)	*P*‐value^b^
Sociodemographics				
Educational level (mother)				
Lower education	1		1	
Intermediate education	0.75 (0.66–0.86)	<0.001	0.73 (0.39–1.37)	0.327
Higher education	0.58 (0.49–0.68)	<0.001	0.68 (0.34–1.37)	0.284
Country of birth (mother)				
The Netherlands	1		1	
Other	1.44 (1.26–1.66)	<0.001	1.44 (0.73–2.86)	0.298
Oral health behaviours				
Age toothbrushing was started				
<1 yr	1		1	
1–2 yr	1.12 (0.99–1.27)	0.069	1.19 (0.69–2.05)	0.535
>2 yr	1.33 (1.07–1.64)	0.009	1.63 (0.62–4.27)	0.324
Toothbrushing frequency				
1 time or less a day	1		1	
2 times a day or more	1.03 (0.90–1.18)	0.699	0.92 (0.51–1.64)	0.777
Family functioning and parenting				
Family functioning – responsiveness				
Normal	1		1	
Subclinical	0.93 (0.74–1.16)	0.504	2.17 (0.81–5.81)	0.122
Clinical	1.68 (1.11–2.55)	0.013	2.77 (0.25–30.5)	0.409
Family functioning – communication				
Normal	1		1	
Subclinical	1.07 (0.90–1.27)	0.447	3.67 (1.60–8.45)	0.002
Clinical	1.43 (1.12–1.83)	0.004	5.52 (1.11–27.3)	0.036
Family functioning – organization				
Normal	1		1	
Subclinical	0.87 (0.72–1.05)	0.141	1.74 (0.82–3.74)	0.151
Clinical	1.44 (1.15–1.82)	0.002	2.88 (0.84–9.88)	0.093
Parenting – restrictiveness				
Low	1		1	
High	1.26 (1.11–1.42)	<0.001	1.44 (0.85–2.45)	0.178
Parenting – nurturance				
Low	1		1	
High	1.03 (0.92–1.16)	0.614	0.54 (0.32–0.90)	0.018

‘1’ is reference category.

a Poisson regression.

b Logistic regression.IRR, incidence risk ratio; OR, odds ratio.

**Figure 2:**
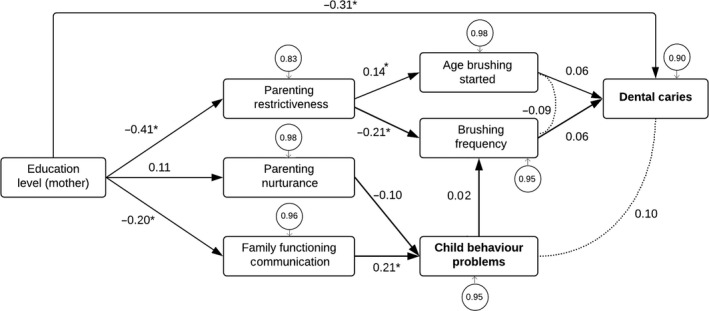
Revised path model. Arrows imply that a variable has an influence on another variable; round connecting lines imply that variables are associated. Values in circles represent unexplained variance. *P < 0.05.

Analysis of the hypothesized path model of Fig. 1 indicated poor fit: *χ* ^2^/d.f. ratio = 147.89/27, *P *<* *0.001; RMSEA = 0.145, 95% CI: 0.123–0.169; SRMR = 0.098, and CFI = 0.428. The model was subsequently modified according to the following steps. Of the family functioning and parenting variables, regression coefficients showed that only restrictiveness remained associated with toothbrushing behaviours, and only nurturance and family functioning, in terms of communication, were associated with externalizing behaviour problems. This implied that retaining only these paths and excluding the responsiveness and organization variables could improve model fit. Regression coefficients also revealed that the mother's country of birth could be omitted when the mother's education level was included. Furthermore, inspection of the standardized residual matrix indicated that the fit could be improved by adding a direct path between the mother's education level and dmft. Although a direct link is evidently not plausible, a conceptual rationale for this modification is that the influence of education level on dental caries acts via several other intermediary variables that were not included in this model. Finally, the model could be improved by adding a correlation between externalizing behaviour problems and dental caries. Application of these modifications resulted in a revised model (Fig. 2), which yielded good fit: *χ* ^2^/d.f. ratio = 18.35/15, *P *=* *0.245; RMSEA = 0.032, 95% CI: 0.000–0.076; SRMR = 0.046, and CFI = 0.964. The paths between externalizing behaviour problems and toothbrushing frequency, and paths between toothbrushing variables and dmft were not statistically significant, yet it was decided to retain these paths for conceptual reasons. The resulting numerical solutions of the revised model were (see Table 4):

**Table 4:** Standardized and unstandardized path coefficients of the revised model

Effects	Standardized path coefficient *(β)*	Unstandardized path coefficient	SE	95% CI	*P‐*value
Dental caries (dmft)					
Education level (mother)	−0.31	−1.35	0.29	−1.92 to −0.79	<0.001
Toothbrushing frequency	0.06	0.42	0.48	−0.53 to 1.37	0.386
Age toothbrushing was started	0.06	0.31	0.34	−0.35 to 0.97	0.351
Toothbrushing frequency					
Parenting – restrictiveness	−0.21	−0.19	0.06	−0.30 to −0.07	0.002
Child behaviour problems	0.02	0.01	0.06	−0.10 to 0.13	0.812
Age toothbrushing was started					
Family functioning – restrictiveness	0.14	0.17	0.09	0.00 to 0.33	0.046
Externalizing behaviour problems					
Parenting – nurturance	−0.10	−0.10	0.07	−0.23 to 0.03	0.120
Family functioning – communication	0.21	0.21	0.07	0.08 to 0.35	0.002
Parenting – restrictiveness					
Education level (mother)	−0.41	−0.28	0.04	−0.36 to −0.20	<0.001
Parenting – nurturance					
Education level (mother)	0.11	0.08	0.05	−0.02 to 0.17	0.106
Family functioning – communication					
Education level (mother)	−0.20	−0.13	0.04	−0.22 to −0.04	0.003

95% CI, 95% confidence interval of the unstandardized path coefficient; dmft, decayed, missing, and filled primary teeth; SE, standard error of the unstandardized path coefficient.

- dmft = (0.06 • age toothbrushing was started) + (0.06 • toothbrushing frequency);
- age toothbrushing was started = 0.14 • parenting restrictiveness;
- toothbrushing frequency = (−0.21 • parenting restrictiveness) + (0.02 • externalizing behaviour problems);
- externalizing behaviour problems = (−0.10 • parenting nurturance) + (0.21 • family functioning communication);
- parenting restrictiveness = −0.41 • education level (mother);
- parenting nurturance = 0.11 • education level (mother); and
- family functioning communication = −0.20 • education level (mother).

## Discussion

This study found that children with elevated or high externalizing behaviour problems had significantly more dental caries experience, but this association did not remain statistically significant after adjustment for sociodemographic characteristics. This study presented a valid model of possible direct and indirect pathways between sociodemographic factors, parenting and family functioning factors, oral health behaviours, externalizing behaviour problems, and dental caries in children. In this model, a lower maternal education level was associated with higher levels of restrictive parenting, lower levels of nurturance, and a higher likelihood of dysfunctional family communication. Higher levels of restrictive parenting were associated with a lower toothbrushing frequency and a higher age at which toothbrushing was started, which, in turn, were associated with greater dental caries experience (but that was not statistically significant). Furthermore, lower levels of nurturance and poorer family functioning in terms of communication were associated with a higher likelihood of externalizing behaviour problems. Externalizing behaviour problems were subsequently linked to dental caries via a direct correlation and through a nonsignificant indirect path via toothbrushing frequency. In addition, lower maternal education was directly associated with greater dental caries experience.

The model suggests that it is plausible that externalizing behaviour problems and dental caries are associated because toothbrushing is more challenging in children with externalizing problem behaviour. The model also provides support for the indirect hypothesis that externalizing behaviour problems and dental caries are associated because family environmental factors, such as low maternal education and negative aspects of parenting and family functioning, coexist in the same families, which may exert an influence on child behaviours in general. However, the inclusion of a direct correlation between behaviour problems and caries in the model indicates that there are other factors that might play a role that are currently not included in the model. For example, it is well known that dietary behaviours are important in the development of dental caries, which could be negatively affected by behavioural problems of the child 22. Adverse structural family characteristics (e.g. single‐parent families), parental stress, negative life events, or conflict at home, are risk factors of the development of both externalizing behaviour problems and dental caries 11, 23, 24. Therefore, the model could be further developed by including the aforementioned factors to improve the model's explanatory power.

This cross‐sectional study permits no conclusions about causality and long‐term findings relating to caries activity. Although variables in the path model were modelled in the sequence of their hypothesized operational order, this ordering does not imply causal effect or provide evidence for temporal precedence of variables. Reciprocal relationships may exist, and the exact role of factors, such as whether they act as mediators, moderators, or confounding factors, cannot be determined. Childhood dental caries is a multifactorial disease that continues to develop as a result of the interplay of different variables. Despite evidence suggesting that parenting and family functioning are stable factors 25, they are subject to all sorts of life events and developments that may affect them over time. Prospective, longitudinal studies are therefore needed to investigate the role of parental, behaviour, and family‐related factors in the initiation of problematic behaviours in children and the development of childhood dental caries over the years. Such studies would allow validation of the model presented in this study, and it would enable further development of the model by including additional explanatory variables.

One strength of the study was that all questionnaires employed were widely used, reliable, and valid instruments 14, 19, 21. Another strength was the way in which children's caries experience was classified. The dmft score was based on dental health records with caries diagnosis supported by dental radiographs. A larger number of approximal lesions can be detected with dental radiographs than by clinical inspection only 26. Bitewings were used in almost all cases, except when children did not wish to cooperate. Children in the study sample were purposively selected from a paediatric dental centre because dental caries and behaviour problems are common conditions in this patient population. As a consequence, the sample was not representative of the Dutch general child population. However, the high levels of caries in the group of children referred (84%) were expected to help explore the research question under consideration because less favourable parenting and behaviour problems could be a determinant of the high caries levels in children. On the other hand, the levels of problem behaviour in the referred children in the practice may have biased the sample: referral is often triggered by treatment failures. Therefore, the high level of caries‐active children could have interfered with differentiation in the caries‐active group in all the constructs measured. In addition, the modest sample size (*n *=* *251) and the relatively high non‐response rate (44%) has resulted in very low numbers of children with poor family functioning. This may have obscured the demonstration of statistically significant associations (type II errors) and also limited the power and the number of variables and paths that could be explored in the structural equation model.

Another limitation is that only one parent, often the mother, completed the questionnaire. Therefore, data on child behavioural problems, family functioning, and parenting solely relied on the view, perceptions, and reporting of one parent. However, the original authors who developed the ‘Family Questionnaire’ evaluated the interparental agreement, and they concluded that mothers and fathers report comparable opinions about their family functioning and parenting 14. This is in line with previously reported literature 27.

The measurement of oral hygiene behaviours using self‐report questionnaires also had its limitations. The oral health behaviours reported in this study may not have been an accurate reflection of actual behaviours because parents could have given socially desirable responses and behaviours were measured at a single point in time, and they can change over the years. Particularly in this sample, children received dental care in a specialized paediatric centre and therefore they probably received oral hygiene instructions and guidance. As a result, oral health behaviours at the time of measurement may have improved since the time that dental caries had developed, or parents may have over‐reported good behaviours. This may explain why no, or only weak, associations between oral hygiene behaviours and dental caries or child behaviour problems were found.

Conceptually, oral health behaviour may play an important role in explaining the relationship between behaviour problems and dental caries. For example, parents may find it more difficult to maintain healthy behaviours if the child shows resistance towards the rules and structures provided by their parents. Communication may also be more challenging in children with externalizing behaviour problems, which could be reflected in the way that parents deal with their children's wishes or demands with regard to – for example – sugary snacks 15.

Children of restrictive parents had significantly greater dental caries experience, in line with findings of previous studies 15, 16. The finding of strict parenting being a risk factor for dental caries is counterintuitive because strictness may be expected to further the establishment of routines, daily structures, and living in accordance with rules. However, similar findings have been found in the literature. Strict, harsh, and coercive parenting is, for example, associated with a higher level of resistance and general noncompliance in children 28. Overly strict and harsh parenting is considered to be negative parenting: associations have also been reported with problem behaviour, childhood obesity, and an unhealthy diet 29. The question that arises is whether negative parenting also makes children less likely to comply with oral health behaviours imposed by the parents.

Poor family functioning in terms of communication and low levels of nurturance were both related to child behavioural problems. These interactions are reciprocal and may create a vicious cycle in which difficult‐to‐manage children elicit more negative and ineffective parental treatment. Less favourable parenting and family functioning may, in turn, contribute to the development of even higher levels of child problem behaviours 30. This bidirectional relationship may have a synergistic effect on the risk of developing dental caries because both are risk factors in the development of the disease and are likely to intensify each other.

In conclusion, this study found that externalizing behaviour problems were associated with greater dental caries experience in children, although this association did not remain statistically significant after adjustment for sociodemographic factors. This study provided some support for a direct path between child behaviour problems and dental caries via toothbrushing behaviour. Yet, the findings also imply that the restrictiveness and warmth of parenting and the communication of the family probably play an indirect role in the association between children's behavioural problems and dental caries experience.

The findings of this study suggest that parent and family factors, such as poor family functioning and strict and harsh parenting, should receive more attention when developing tailored caries‐preventive approaches, particularly when children have behaviour problems. The reciprocal association between family factors and child behaviour problems should be considered because this may be a complicating factor in establishing dentally healthy behaviours. Given the ineffectiveness of health education by teaching knowledge alone, the needs of the patient and the family as a whole should be considered. More research is needed to evaluate preventive interventions that target these factors. Education for dental students is lacking in this field, so in the event that positive findings are identified in prospective interventions, a paradigm shift will be required to educate a new generation and to introduce the consideration of these factors into daily practice.

## Conflicts of interest

The authors declare that they have no conflict of interest.

## References

[1] Sluis S , Polderman TJ , Neale MC , Verhulst FC , Posthuma D , Dieleman GC . Sex differences and gender‐invariance of mother‐reported childhood problem behavior. Int J Meth Psychiatr Res 2017; 26: e1498 10.1002/mpr.1498 PMC6877260 26799863

[2] Bor W , Dean AJ , Najman J , Hayatbakhsh R . Are child and adolescent mental health problems increasing in the 21st century? A systematic review. Aust N Z J Psychiatr 2014; 48: 606–616. 10.1177/0004867414533834 24829198

[3] Liu J . Childhood externalizing behavior: theory and implications. J Child Adolesc Psychiatr Nurs 2004; 17: 93–103. 15535385 10.1111/j.1744-6171.2004.tb00003.x PMC1617081

[4] Williamson R , Oueis H , Casamassimo PS , Thikkurissy S . Association between early childhood caries and behavior as measured by the child behavior checklist. Pediatr Dent 2008; 30: 505–509. 19186777

[5] Blomqvist M , Ahadi S , Fernell E , Ek U , Dahllöf G . Dental caries in adolescents with attention deficit hyperactivity disorder: a population‐based follow‐up study. Eur J Oral Sci 2011; 119: 381–385. 21896055 10.1111/j.1600-0722.2011.00844.x

[6] Broadbent JM , Ayers KM , Thomson WM . Is attention‐deficit hyperactivity disorder a risk factor for dental caries? A case‐control study Caries Res 2004; 38: 29–33. 14684974 10.1159/000073917

[7] Holmberg K , Fernell E , Ek U , Dahllöf G . Dental caries and oral health behavior in children with attention deficit hyperactivity disorder. Eur J Oral Sci 2007; 115: 186–191. 17587293 10.1111/j.1600-0722.2007.00451.x

[8] Hidas A , Noy AF , Birman N , Shapira J , Matot I , Steinberg D , Moskovitz M . Oral health status, salivary flow rate and salivary quality in children, adolescents and young adults with ADHD. Arch Oral Biol 2011; 56: 1137–1141. 21514566 10.1016/j.archoralbio.2011.03.018

[9] Nanjappa S , Hector M , Marcenes W . Mother's perception of general family functioning and sugar consumption of 3‐and 4‐year‐old children: the East London family study. Caries Res 2015; 49: 515–522. 26304625 10.1159/000431234

[10] Kremers SP , Brug J , de Vries H , Engels RC . Parenting style and adolescent fruit consumption. Appetite 2003; 41: 43–50. 12880620 10.1016/s0195-6663(03)00038-2

[11] Hooley M , Skouteris H , Boganin C , Satur J , Kilpatrick N . Parental influence and the development of dental caries in children aged 0–6 years: a systematic review of the literature. J Dent 2012; 4: 873–885. 10.1016/j.jdent.2012.07.013 22842202

[12] Bosmans G , Braet C , van Leeuwen K , Beyers W . Do parenting behaviors predict externalizing behavior in adolescence, or is attachment the neglected 3rd factor? J Youth Adolesc 2006; 35: 354–364.

[13] Maccoby EE , Martin JA . Socialization in the context of the family: parent‐child interaction. Handbook of child psychology, 4th edn Chichester, NY: Wiley, 1983.

[14] van der Ploeg JD , Scholte EM . Handleiding Gezinsvragenlijst (GVL). Houten: Bohn Stefleu van Loghum, 2008.

[15] Howenstein J , Kumar A , Casamassimo PS , McTigue D , Coury D , Yin H . Correlating parenting styles with child behavior and caries. Pediatr Dent 2015; 37: 59–64. 25685975 PMC4559268

[16] De Jong‐Lenters M , Duijster D , Bruist M , Thijssen J , de Ruiter C . The relationship between parenting, family interaction and childhood dental caries: a case‐control study. Soc Sci Med 2014; 116: 49–55. 24980791 10.1016/j.socscimed.2014.06.031

[17] Abegg C , Croucher R , Marcenes WS , Sheiham A . How do routines of daily activities and flexibility of daily activities affect tooth‐cleaning behavior? J Public Health Dent 2000; 60: 154–158. 11109212 10.1111/j.1752-7325.2000.tb03321.x

[18] Duijster D , Verrips GHW , Loveren C . The role of family functioning in childhood dental caries. Community Dent Oral Epidemiol 2014; 42: 193–205. 24117838 10.1111/cdoe.12079

[19] Goodman R . Psychometric properties of the strengths and difficulties questionnaire. J Am Acad Child Adolesc Psychiatr 2001; 40: 1337–1345. 10.1097/00004583-200111000-00015 11699809

[20] http://www.sdqinfo.com. Accessed on April 2016.

[21] Deković M , Janssens JM , Gerris JR . Factor structure and construct validity of the Block Child Rearing Practices Report (CRPR). Psychological Assessment J Consult Clin Psychol 1991; 3: 182.

[22] Harris R , Nicoll AD , Adair PM , Pine CM . Risk factors for dental caries in young children: a systematic review of the literature. Community Dent Health 2004; 21(supplement): 71–85. 15072476

[23] Deater–Deckard K , Dodge KA , Bates JE , Pettit GS . Multiple risk factors in the development of externalizing behavior problems: Group and individual differences. Dev Psychopathol 1998; 10: 469–493. 9741678 10.1017/s0954579498001709 PMC2776047

[24] Nelson S , Lee W , Albert JM , Singer LT . Early maternal psychosocial factors are predictors for adolescent caries. J Dent Res 2012; 91: 859–864. 22821239 10.1177/0022034512454434 PMC3420393

[25] Shaffer A , Lindhiem O , Kolko DJ , Trentacosta CJ . Bidirectional relations between parenting practices and child externalizing behavior: a cross‐lagged panel analysis in the context of a psychosocial treatment and 3‐year follow‐up. J Abnorm Child Psychol 2013; 41: 199–210. 22821450 10.1007/s10802-012-9670-3 PMC3626089

[26] Poorterman JH , Vermaire EH , Hoogstraten J . Value of bitewing radiographs for detecting approximal caries in 6‐year‐old children in the Netherlands. Int J Paediatr Dent 2010; 20: 336–340. 20545787 10.1111/j.1365-263X.2010.01058.x

[27] Landis JR , Koch GG . The measurement of observer agreement for categorical data. Biometrics 1977; 33: 159–174. 843571

[28] Kuczynski L , Kochanska G , Radke‐Yarrow M , Girnius‐Brown O . A developmental interpretation of young children's noncompliance. Dev Psychol 1987; 23: 799.

[29] Rhee K . Childhood overweight and the relationship between parent behaviors, parenting style, and family functioning. Ann Am Acad Pol Soc Sci 2008; 615: 11–37.

[30] Hipwell A , Keenan K , Kasza K , Loeber R , Stouthamer‐Loeber M , Bean T . Reciprocal influences between girls’ conduct problems and depression, and parental punishment and warmth: a six year prospective analysis. J Abnorm Child Psychol 2008; 36: 663–677. 18172753 10.1007/s10802-007-9206-4 PMC2572202

